# Superior Mesenteric Artery Syndrome with Abdominal Compartment Syndrome

**DOI:** 10.1155/2016/7809281

**Published:** 2016-11-27

**Authors:** Kevin Reece, Rachel Day, Janna Welch

**Affiliations:** University of Texas Dell Medical School, Emergency Medicine Residency, Austin, TX, USA

## Abstract

Superior Mesenteric Artery (SMA) syndrome is a condition in which the duodenum becomes compressed between the SMA and the aorta, resulting in bowel obstruction which subsequently compresses surrounding structures. Pressure on the inferior vena cava (IVC) and aorta decreases cardiac output which compromises distal blood flow, resulting in abdominal compartment syndrome with ischemia and renal failure. A 15-year-old male with SMA syndrome presented with 12 hours of pain, a distended, rigid abdomen, mottled skin below the waist, and decreased motor and sensory function in the lower extremities. Exploratory laparotomy revealed ischemic small bowel and stomach with abdominal compartment syndrome. Despite decompression, the patient arrested from hyperkalemia following reperfusion.

## 1. Introduction

Superior Mesenteric Artery syndrome is a rare but potentially life threatening condition in which the duodenum becomes compressed between the SMA and the aorta. Early symptoms can include simply nausea and early satiety. However, obstruction can progress rapidly and severely, resulting in compression of the inferior vena cava and aorta [1]. Subsequent decreases in cardiac output and distal ischemia cause lactic acidosis, metabolic derangement, and ultimately death.

## 2. Case Report

A 15-year-old Hispanic male presented with severe abdominal pain and distention that progressively worsened over the past 12 hours. The patient was reportedly asymptomatic the day before. He also complained of pain and difficulty moving his legs bilaterally. He had no associated vomiting, diarrhea, or fever.

**Figure 1:**
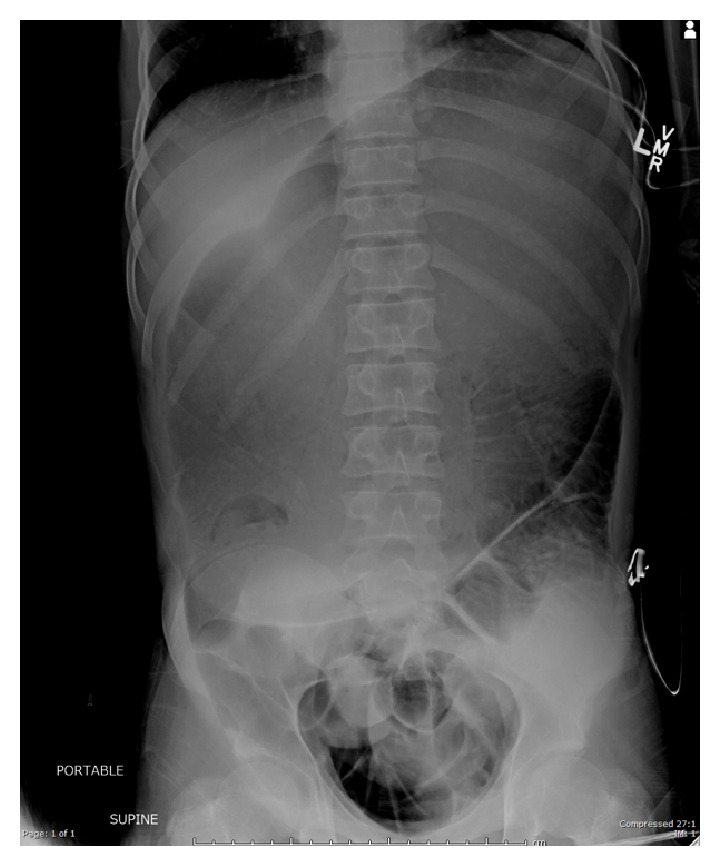
Obtained from patient medical records with permission granted by Seton Family of Hospitals, September 2016.

The patient's mother reported a past medical history of premature birth, developmental delay, and a recent diagnosis of Superior Mesenteric Artery syndrome. The diagnosis of SMA syndrome was made after similar episode of abdominal pain and distention 4 months earlier. According to inpatient records, the patient received nasojejunal tube decompression followed by tube feeds to increase the size of his mesenteric fat pad. No surgical repair was scheduled. The patient's mother denied any other symptoms since that episode (Figure 1).

On physical exam, the patient was alert and in severe distress. His heart rate was 150 beats/min with a blood pressure of 105/75 mmHg. He was tachypneic with clear lung sounds. His abdomen was massively distended, rigid, and diffusely tender to palpation. His skin was cold and mottled from the waist down and he had decreased motor function and sensation in his legs bilaterally. Femoral pulses were not palpable, but radial pulses were present.

Abdominal X-ray showed massive distention of the stomach without free air. Venous blood gas showed pH 6.9, bicarbonate level 17, and lactate 10.7. Complete blood count showed a white blood cell count of 22000, hemoglobin of 19.3, hematocrit of 57.9, and platelets of 288. His metabolic panel was notable for carbon dioxide level of 14, anion gap of 29, BUN of 24, and creatinine of 2.4. Liver function tests were within normal limits.

The patient was given a 30 mL/kg normal saline bolus. An attempt was made to pass a nasogastric tube for gastric decompression, but the tube could not be advanced past the gastroesophageal junction. Based on clinical exam, severe abdominal compartment syndrome with compression of arterial flow through the aorta was suspected. In consultation with the surgery team, the decision was made to take the patient for emergent decompressive laparotomy without delaying care further for compartment pressures testing. Emergent needle decompression of the stomach would have been another option for the emergency physician if the operating room had not been immediately available or if care was being provided at an outlying facility and transport was indicated. The surgical team wanted to avoid any delay to surgery. The patient was in the operating suite within one hour of arrival to the hospital.

In anticipation of significant reperfusion injury, the patient was given calcium gluconate, sodium bicarbonate, insulin, and dextrose at the start of surgery. Upon opening the abdomen, the patient was noted to have a massively distended stomach with an ischemic appearance. The small bowel was also noted to be dusky from ischemia. The stomach was incised and a large quantity of air and partially digested food was evacuated. With decompression of the stomach, the patient had return of femoral pulses and the lower extremities regained color.

Shortly after decompression of the stomach, the patient's arterial blood gas showed a pH of 6.7, HCO_3_ of 10, lactate of 13, and potassium of 8.7. Additional bicarbonate, insulin, and dextrose were given. The patient then suffered cardiac arrest second to hyperkalemia and underwent an hour of Advanced Cardiac Life Support (ACLS) protocol resuscitation in the operating room. During the code, the patient's lactate climbed to 15 and the potassium fluctuated between 7 and 8 on multiple serial blood gases despite medical intervention to stabilize it. The patient regained pulses and was moved to the intensive care unit (ICU).

In the ICU, the patient was started on continuous dialysis. Despite resuscitation efforts including high doses of vasopressors to maintain blood pressure as well as sodium bicarbonate, insulin, glucose, albuterol, and calcium, the patient suffered another episode of cardiac arrest. Despite resuming ACLS protocol, the patient was unable to be resuscitated. The patient expired in the intensive care unit 5 hours after arrival to the emergency department.

## 3. Discussion

Superior Mesenteric Artery syndrome, although rare, should be on the emergency physician's differential when assessing a patient with signs and symptoms of intestinal obstruction. It is generally thought of as a diagnosis of exclusion.

The mechanism of obstruction in SMA syndrome involves the third portion of the duodenum becoming compressed between the SMA and the aorta. It is thought to be due to a loss of the mesenteric fat pad that usually keeps these structures apart. The angle created by the SMA and the aorta in a normal individual is between 38 and 65 degrees [2]. As this angle becomes more acute, there is a higher risk for SMA syndrome and resulting bowel obstruction.

SMA syndrome is unlikely in young healthy adults and children. It generally affects individuals with underlying medical comorbidities, such as AIDS, malignancy, or even patients that have undergone extreme weight loss after bariatric surgery.

It is speculated that there is a genetic predisposition to SMA syndrome. Some individuals are born with a congenitally shortened ligament of Treitz—the suspending ligament of the duodenum. This results in suspension of the duodenum more cephalad than usual—closer to the SMA's origin from the aorta where compression is more likely [3].

As with all cases of bowel obstruction, patient presentation can be acute or more insidious. Typical symptoms of bowel obstruction such as abdominal pain and distention, nausea and vomiting, and obstipation can all be expected but are not the rule. Those with a partial obstruction may only complain of nausea after eating and early satiety. Patients may experience relief when lying prone, as opposed to supine—where gravity is contributing to the duodenal compression [4]. According to a 22-case review of pediatric patients diagnosed with SMA syndrome at a children's hospital in Wisconsin, “presenting symptoms included: abdominal pain (59%), vomiting (50%), nausea (40%), early satiety (32%) and anorexia (18%)” [5]. The differential diagnosis should include all causes of bowel obstruction as well as causes of gastrointestinal dysmotility.

Even with advances in diagnostic imaging, in the pediatric patient, physical exam remains the modality of choice for diagnosis [6]. A plain abdominal film should also be ordered to confirm clinical suspicion. In the review of 22 pediatric cases in Wisconsin, diagnosis was confirmed by upper-GI radiography in 18 (82%), by CT in 2 (9%), and at laparotomy in 2 (9%) [5]. Plain radiograph may reveal evidence of proximal obstruction such as massive gastric distention, as was the case with our patient. Those proficient with ultrasound can choose to measure the aortomesenteric angle, which may give clues pointing towards SMA syndrome if the angle is more acute. An angle of less than 25% is concerning for SMA syndrome [7].

In the pediatric population, conservative management includes nutritional support to encourage weight gain and growth of the fat pad between the SMA and aorta [6]. Oral intake is ideal but not always achievable. For those unable to attain adequate caloric intake by mouth, NJ tube feedings placed past the obstruction can be used. Total parenteral nutrition is also an option if NJ feeds remain inadequate. Nutritional support is usually sufficient treatment, and surgical management is considered when disease remains refractory to this. One such operation, called Strong's procedure, looks to mobilize the duodenum from the ligament of Treitz and place it to the right of the SMA [8]. Strong's procedure is considered the most conservative surgical management in the pediatric population [6]. Another option is a gastrojejunostomy—where the jejunum is connected directly to the stomach, bypassing the duodenum entirely. When patients are medically or surgically stabilized, they are encouraged to eat small frequent meals to avoid significant obstruction. The mother of our patient reported our patient consuming a large high calorie Chinese food meal the night prior to presentation, which likely contributed to the severity of his symptoms.

The treatment of acute decompensated SMA syndrome in the crashing patient does not offer the luxury of time. As with any cause of acute bowel obstruction, elimination of symptoms and resolution of obstruction are the goal. An NG tube should be placed to low intermittent suction. Unfortunately, in this case, a nasogastric tube could not be successfully placed in the emergency department, even in the hands of experienced surgeons. Electrolyte abnormalities due to vomiting should be corrected and fluid resuscitation should be initiated. Further supportive care should be dictated by patient presentation and clinical picture.

Our patient ultimately required an emergent exploratory laparotomy and gastric decompression. As the patient was in extremis, a joint decision was made with the surgical team that he would not survive conservative management and would require emergent operative care. His obstruction was so severe that he had presumably developed an abdominal compartment syndrome with ischemia of his intra-abdominal organs as well as his lower extremities. This diagnosis was made based on exam and was confirmed with intraoperative findings. An abdominal compartment pressure was never measured in this patient but was deemed unnecessary based on exam and severity of presentation. The abdominal compartment syndrome that developed secondary to his severe SMA syndrome is a complex and difficult to manage disease process that is described briefly below.

The textbook definition of abdominal compartment syndrome is sustained intra-abdominal pressures exceeding 20 mmHg [1]. Abdominal compartment syndrome can be diagnosed at lower pressures if there are signs of new organ dysfunction. The abdominal wall compliance initially helps minimize the effects of the increasing pressure, as abdominal girth can increase to a certain degree. When abdominal wall compliance reaches its critical limit, the abdominal compartment pressure will rapidly rise [9]. This helps explain why our patient became so severely symptomatic so acutely.

The consequences of abdominal compartment syndrome can affect nearly every system in the body. Cardiac output is decreased secondary to reduced venous filling as the IVC is compressed. There is reduced chest wall compliance and smaller tidal volumes, leading to hypoxemia and hypercarbia [10]. Renal failure occurs due to decreased renal arterial blood flow, as well as renal congestion from impaired venous drainage secondary to renal vein compression. The bowel becomes ischemic, causing a lactic acidosis. Loss of the intestinal mucosal barrier can allow for bacterial translocation and sepsis. The liver is unable to clear the increasing lactic acid at pressures as small as 10 mmHg [11].

Ultimately, after relief of the obstruction in the operating room, reperfusion injuries were presumably responsible for severe acidosis and electrolyte abnormalities that were not compatible with life—even with aggressive resuscitation and emergent dialysis. The patient's hyperkalemia was not responsive to usual measures and his lactate continued to rise. Ischemic reperfusion injury occurs at the cellular level. During ischemia, reactive nitrogen species build up inside cells. These ultimately become reactive oxygen species upon reintroduction of oxygen to the ischemic cells [12]. Cell death and apoptosis are the result of excessive levels of reactive oxygen species. Reperfusion paradoxically leads to more cell death than ischemia alone. Potassium is released from ischemic muscles and upon reperfusion is mobilized to the systemic circulation. The heart is especially susceptible to the elevated potassium levels with feared complications of bradycardia and possibly cardiac arrest.

The patient ultimately suffered from cardiac arrest due to the severe metabolic derangements, notably a refractory hyperkalemia from reperfusion despite aggressive medical management and prompt initiation of emergency dialysis. This was a difficult case to manage of a rare disease entity. The surgeons and anesthesiologist were well aware of the consequences that would come with the decompression of obstruction and resolution of abdominal compartment syndrome. In fact, prior to decompression, the surgeons notified anesthesia that they were about to see the effects of the reperfusion injury—soon after the patient did indeed arrest. Could something have been done in the emergency department or surgical suite that would have saved this patient's life? The time from initial presentation to the emergency department to first incision was under 60 minutes. The patient received maximal medical resuscitation to combat the metabolic derangements. Dialysis could have possibly been initiated sooner; however, the patient had already been coded for an hour prior to leaving the OR and his prognosis was dismal. Nasogastric tube placement in the ED would have been optimal, but even in experienced hands it was not possible—both secondary to patient compliance and severity of presentation. Ideally, this patient would have presented much sooner in his acutely decompensated course, when medical management could offer a better chance at resolution prior to onset of abdominal compartment syndrome and subsequent reperfusion injury leading to cardiac arrest.

## References

[1] Malbrain M. L. N. G., Cheatham M. L., Kirkpatrick A. (2006). Results from the international conference of experts on intra-abdominal hypertension and abdominal compartment syndrome. I. Definitions. *Intensive Care Medicine*.

[2] Ozkurt H., Cenker M. M., Bas N., Erturk S. M., Basak M. (2007). Measurement of the distance and angle between the aorta and superior mesenteric artery: normal values in different BMI categories. *Surgical and Radiologic Anatomy*.

[3] Iwaoka Y., Yamada M., Takehira Y. (2001). Superior mesenteric artery syndrome in identical twin brothers. *Internal Medicine*.

[4] Wilkie D. P. (1921). Chronic duodenal ileus. *British Journal of Surgery*.

[5] Biank V., Werlin S. (2006). Superior mesenteric artery syndrome in children: a 20-year experience. *Journal of Pediatric Gastroenterology and Nutrition*.

[6] Record J. L., Morris B. G., Adolph V. R. (2015). Resolution of refractory superior mesenteric artery syndrome with laparoscopic duodenojejunostomy: pediatric case series with spectrum of clinical imaging. *Ochsner Journal*.

[7] Neri S., Signorelli S. S., Mondati E. (2005). Ultrasound imaging in diagnosis of superior mesenteric artery syndrome. *Journal of Internal Medicine*.

[8] Wilson-Storey D., MacKinlay G. A. (1986). The superior mesenteric artery syndrome. *Journal of the Royal College of Surgeons of Edinburgh*.

[9] Vidal M. G., Weisser J. R., Gonzalez F. (2008). Incidence and clinical effects of intra-abdominal hypertension in critically ill patients. *Critical Care Medicine*.

[10] Cullen D. J., Coyle J. P., Teplick R., Long M. C. (1989). Cardiovascular, pulmonary, and renal effects of massively increased intra-abdominal pressure in critically ill patients. *Critical Care Medicine*.

[11] Luca A., Cirera I., García-Pagán J. C. (1993). Hemodynamic effects of acute changes in intra-abdominal pressure in patients with cirrhosis. *Gastroenterology*.

[12] Kalogeris T., Baines C. P., Krenz M., Korthuis R. J. (2014). Chapter six—cell biology of ischemia/reperfusion injury. *International Review of Cell and Molecular Biology*.

